# Modeling HCV disease in animals: virology, immunology and pathogenesis of HCV and GBV-B infections

**DOI:** 10.3389/fmicb.2014.00690

**Published:** 2014-12-08

**Authors:** Cordelia Manickam, R. Keith Reeves

**Affiliations:** Center for Virology and Vaccine Research, Beth Israel Deaconess Medical Center – Harvard Medical SchoolBoston, MA, USA

**Keywords:** GBV-B, HCV, non-human primate, marmoset, animal models

## Abstract

Hepatitis C virus (HCV) infection has become a global public health burden costing billions of dollars in health care annually. Even with rapidly advancing scientific technologies this disease still poses a significant threat due to a lack of vaccines and affordable treatment options. The immune correlates of protection and predisposing factors toward chronicity remain major obstacles to development of HCV vaccines and immunotherapeutics due, at least in part, to lack of a tangible infection animal model. This review discusses the currently available animal models for HCV disease with a primary focus on GB virus B (GBV-B) infection of New World primates that recapitulates the dual *Hepacivirus* phenotypes of acute viral clearance and chronic pathologic disease. HCV and GBV-B are also closely phylogenetically related and advances in characterization of the immune systems of New World primates have already led to the use of this model for drug testing and vaccine trials. Herein, we discuss the benefits and caveats of the GBV-B infection model and discuss potential avenues for future development of novel vaccines and immunotherapies.

Hepatitis C virus (HCV) is a major chronic disease that has infected greater than 150 million people worldwide. Given the breadth and duration of infection HCV disease could result in an economic crisis reaching into the 100s of billions within the next decade. Although acute infection is usually asymptomatic with self-resolution, 55–85% of infected persons develop chronic disease that can lead to progressive hepatic fibrosis, cirrhosis, and hepatocellular carcinoma (HCC; Hoofnagle, 2002) which is often fatal without treatment. There is also a strong association of HCV infection with extra-hepatic diseases such as cryoglobulinemia vasculitis (Luppi et al., 1998), diabetes (Mehta et al., 2000), thyroid disease (Pateron et al., 1992), lichen planus (Jubert et al., 1994), and neuropsychiatric conditions such as depression (Thomas, 2013). This is complicated by the fact that approximately only half of individuals are aware of their HCV status (Denniston et al., 2012), due, at least in part, to the rarity of symptoms in acute HCV infection. In 2007, mortality due to HCV infection surpassed that of HIV in the United States (Ly et al., 2012).

## HCV DISEASE IN HUMANS

Hepatitis C virus was first identified by Choo et al. (1989) as the etiologic agent of non-A, non-B hepatitis. HCV is an enveloped, single-stranded, positive sense RNA virus belonging to the *Hepacivirus* genus within the *Flaviviridae* family. The genome is 9.6 kb with a single open reading frame (ORF) that encodes a polyprotein which is further cleaved to yield 10 mature viral proteins namely the structural proteins (C, E1, E2) and non-structural proteins (p7, NS2, NS3, NS4A, NS4B, NS5A, NS5B; Hellen and Pestova, 1999; Lohmann, 2013). The envelope proteins (E1 and E2) and NS1 are encoded from the most variable regions of the HCV genome while NS5B codes for the RNA-dependent RNA polymerase (RdRp) that is more highly conserved. HCV strains are classified into seven genotypes and 67 subtypes (Smith et al., 2014) based on sequence heterogeneity. Genotypes 1 and 2 are distributed globally while genotype 3 predominates in Southeast Asia, genotype 4 in most of Africa, genotype 5 in South Africa, and genotype 6 primarily in Hong Kong and Vietnam (Gower et al., 2014). A genotype 7 variant has been confirmed in only one Canadian patient who had emigrated from the Democratic republic of Congo (Murphy et al., 2007; Smith et al., 2014). In the US, 70% of HCV infections are due to genotype 1 (Gower et al., 2014). Transmission of HCV occurs primarily through exposure to HCV-infected blood with an average incubation period of 6–10 weeks (Alter et al., 1989; Barrera et al., 1995). Increased alanine aminotransferase (ALT) levels, as a marker for liver damage, are also observed within 8–12 weeks after exposure (Rehermann, 2009). Many individuals clear the virus within 6–12 months, but the outcome of the infection depends on multiple factors including host genetics, virus strain, and virus-specific immunity (Abdel-Hakeem and Shoukry, 2014). Ethnicity, sex, alcohol abuse, and co-infections can also contribute to the outcome of infection (Thomas et al., 2000; Schiff and Ozden, 2003; Baden et al., 2014). However, the full spectrum of factors that dictate clearance versus chronicity remains unknown.

Traditionally, therapy for chronic HCV has utilized a combination of pegylated-IFN-α and ribavirin (Manns et al., 2001; Fried et al., 2002), and later direct-acting antivirals Boceprevir and Telaprevir in combination with pegylated IFN-α and ribavirin (Jacobson et al., 2011; Poordad et al., 2011). Recently, Sofosbuvir, a nucleotide analog HCV NS5B polymerase inhibitor developed by Gilead, has been approved as part of an antiviral regimen (Keating and Vaidya, 2014). This represents a breakthrough in HCV therapeutics since Sofosbuvir is a simplified once-daily oral treatment (Cholongitas and Papatheodoridis, 2014; Keating and Vaidya, 2014). However, the high cost and sophisticated clinical monitoring required will likely make implementation challenging in developing countries.

Despite two decades of research, no vaccine for HCV is available. Several candidate vaccines that reached the stage of Phase I or II clinical trials did not progress further (Xue et al., 2014; Zingaretti et al., 2014). One of the major challenges (summarized in **Table 1**) for vaccine development is the lack of small animal models that would allow the study of HCV infection, genetic variability of host defenses, and immune modulatory mechanisms induced by the virus. GBV-B is phylogenetically a close relative of HCV and infects only New World monkeys such as tamarins and marmosets (Muerhoff et al., 1995; Ohba et al., 1996). Although GBV-B causes acute hepatitis with few cases of persistence, it has been used as a surrogate model for HCV infection in various studies to understand pathogenesis and to demonstrate candidate antiviral activity (Beames et al., 2000, 2001; Bright et al., 2004; Iwasaki et al., 2011). Even the differential infection profiles between the two viruses could prove helpful in grasping the immune evasion strategies of HCV. For example, the HCV non-structural protein 5A (NS5A), implicated in establishing persistence, modulates the Ras-Erk signaling pathway (Mankouri et al., 2008a), whereas the GBV-B NS5A protein does not block this pathway and also differs in its cellular distribution (Mankouri et al., 2008b). This suggests that the altered mechanism of NS5A in HCV infection could be responsible for the chronicity of HCV in humans. Thus, both the similarities and the contrasts of the GBV-B model with the HCV infection can serve to broaden the knowledge of HCV pathogenesis.

**Table 1:** Challenges in study and control of HCV.

Genetic diversity of the virus	Genotypes are at least 30% divergent in nucleotide sequence Quasispecies variation introduced by error prone RNA dependent RNA polymerase (RdRp)
Propagation of virus *in vitro*	Difficulty in isolation and propagation of HCV in cell lines
Vaccine development	Limited human studies in HCV at-risk populations Lack of suitable animal models to understand viral pathogenesis and execute vaccine studies
Treatment	Expensive, limited availability

## HISTORY OF GBV-B

In an attempt to isolate and identify the etiologic agent(s) responsible for non-A, non-B hepatitis, Deinhardt et al. (1967) inoculated tamarins and marmosets with the sera from patients with severe hepatitis. The serum from one individual, a surgeon whose initials were GB, was found to cause hepatitis in four challenged tamarins. Serial passages of the infected tamarin sera also caused hepatitis in some animals. The 11th passage of this serum (H205 GB pass 11 serum) was demonstrated to be infectious in most tamarins and later was used as the primary virus source for subsequent studies (Deinhardt et al., 1975; Muerhoff et al., 1995; Simons et al., 1995b; Bukh et al., 1999, 2001). Interestingly, the H205 GB pass 11 serum was found to be non-infectious in chimpanzees (Tabor et al., 1980). Reciprocally, tamarins were not infected by HCV (Garson et al., 1997) implying that the GB infectious agent and HCV are different entities.

In Simons et al. (1995b) two viruses were identified from the serum of a tamarin infected with the H205 GB pass 11 serum and were termed GBV-A and GVB-B. The genomic structure of both the GB agents were similar to the *Flaviviridae* family of viruses. GBV-B in particular was found to be closely related to HCV based on amino acid sequence alignments and phylogenetic analyses (**Table 2**; Muerhoff et al., 1995). A third virus, GBV-C, was also identified in human serum samples by the usage of degenerate oligonucleotides that amplify related viral sequences (Simons et al., 1995a). GBV-C was later determined to be the same virus as hepatitis G virus (HGV; Linnen et al., 1996). GBV-A was identified as a common primate virus with viral genomes identified in *Sanguinus*, *Callithrix,* and *Aotus* species (Bukh and Apgar, 1997). GBV-A does not appear to cause hepatitis, but can persistently infect New World monkeys with no obvious disease symptoms (Bukh and Apgar, 1997). Phylogenetic analysis showed that HCV and GBV-B were closely related while GBV-A and GBV-C form a separate cluster (Muerhoff et al., 1995; Simons et al., 1995a). The GBV-A and GBV-C viruses have now been classified under a different genus, *Pegivirus*, and renamed as simian pegivirus (SPgV) and human pegivirus (HPgV) respectively (Stapleton et al., 2011). Unlike GBV-A, GBV-B is not commonly found in New World monkeys and its natural host range is still unknown. Experimental inoculation with GBV-B results in elevated liver enzymes in tamarins and can afford protection against GBV-B re-infection (Schaluder et al., 1995). Further, development of acute hepatitis in tamarins by transfection studies with RNA transcripts of an infectious clone of GBV-B confirmed the hepatotropism of GBV-B (Bukh et al., 1999).

**Table 2:** Hepatitis C virus and related flaviviruses.

	Nucleotide sequence homology to HCV based on NS3 and NS5b genes	Disease characteristics
**Hepacivirus**
GBV-B	48–62% (Muerhoff et al., 1995)	Infects New World monkeys; hepatotrophic; acute hepatitis; spontaneous clearance in most cases (Stapleton et al., 2011)
CHV	50% (Kapoor et al., 2011; Pfaender et al., 2014)	Infects dogs; not associated with liver disease; virus found in nasal samples
NHPV (CHV-like virus)		Infects horses; associated with acute and chronic infection of liver (Pfaender et al., 2014)
**Pegivirus**
GBV-A	53–55% (Muerhoff et al., 1995)	Infects New World monkeys; lymphotrophic; life-long infection; no apparent disease (Stapleton et al., 2011)
GBV-C	40–50% (Linnen et al., 1996)	Infects humans; lymphotrophic; persistent infection; no evidence of liver damage (Stapleton et al., 2011)
GBV-D	41% (Epstein et al., 2010)	Infects bats; no apparent hepatic dysfunction (Epstein et al., 2010)

## OTHER HEPACIVIRUSES

The origin of HCV in humans is still not completely understood as no HCV or HCV-like viruses have been discovered in Old World monkeys. To understand the evolutionary origin of HCV and to identify and characterize animal viral homologs (**Table 2**), the sera of multiple non-primate species were screened for the presence of hepaciviruses (Burbelo et al., 2012). Canine hepacivirus (CHV), which has just 50% nucleotide sequence divergence from HCV, was found to cause a respiratory disease in dogs associated with high virus replication in the respiratory tract (Kapoor et al., 2011). Serological analysis revealed the presence of virus variants similar to CHV in horses, termed as non-primate hepaciviruses (NPHVs), with rates of persistence greater than 20% and that cluster with the canine/equine clade (Burbelo et al., 2012). Acute and chronic hepatitis has been associated with NHPV infection in horses with greater than 60% clearance of infection within first 2 months of infection (Pfaender et al., 2014). Novel hepaciviruses have also been isolated from European bank voles and South African four-striped grass mice and although these viruses are related to GBV-B their ability to cause disease remains to be determined (Drexler et al., 2013).

## MODELS OF HCV INFECTION

### CHIMPANZEE

Chimpanzees (*Pan troglodytes*) were the first animal model for HCV infection and have contributed significantly to understanding the natural history of HCV (Houghton, 2009; **Table 3**). Chimpanzees also played a critical role in discovery of HCV (Alter et al., 1978), and in the absence of efficient cell culture systems, most infectious clones of HCV were developed *in vivo* (Kolykhalov et al., 1997; Yanagi et al., 1997). Chimpanzees have also been the primary model for evaluation of various antivirals and vaccine modalities (Choo et al., 1994; Folgori et al., 2006; Carroll et al., 2009; Summa et al., 2012; Callendret et al., 2014). Similar to HCV infection in humans, infection of chimpanzees can result in either an acutely cleared disease or chronic infection (Walker, 1997; Major et al., 2004). Acute infection is characterized by early appearance of viremia (within a week) reaching peak viral titers of 10^5^–10^7^ genome copies/ml in plasma (Walker, 1997; Bukh, 2004). Antibodies against HCV protein are generally detected within the first 2 months of infection (Bukh, 2004). Hepatitis is evidenced by elevated serum liver enzyme values and necroinflammatory changes in the liver. Approximately 60% of infected chimpanzees develop chronic disease (Abe et al., 1992), characterized by persistent viremia and mild hepatitis. Unfortunately, the use of chimpanzees as an animal model for HCV continues to be limited due to their low availability, high expense, ethical concerns, and a disease course significantly attenuated compared to humans (Bukh, 2004).

**Table 3:** Animal models for HCV.

Model	Advantages	Limitations
Chimpanzee	HCV discovery; *in vivo* virus replication	Expensive; limited availability; lack of liver fibrosis; low chronicity rate; ethical concerns
Tree shrew	Small animal model susceptible to HCV infection	Lack of chronicity; only transient viremia
Humanized mice model	Useful for immunization and challenge studies	Low level of viral replication; lack of progressive liver pathology
GBV-B infection of New World monkeys	GBV-B closely related to HCV; analogous disease course to HCV	Low frequency of chronic disease
Chimeric GBV-B infection of New World monkeys	Antiviral testing	Inability to cause chronic infection

### TREE SHREW

The northern tree shrew (*Tupaia belangeri*) is a non-rodent small mammal indigenous to Southeast Asia, and other than chimpanzees and humans, tree shrews are the only other species known to be susceptible to HCV infection (Xie et al., 1998). In a 3-year longitudinal study (Amako et al., 2010) persistent liver infection was found in some animals and included some histological indications of liver disease such as steatosis, fibrosis, and cirrhosis. Viremia and serum antibody levels were intermittent, and in general the course of infection was transient and self-resolving. Although infected tree shrews have been used for metabolomic analyses to identify biomarkers associated with HCV infection (Sun et al., 2013), the limited virus replication and lack of species-specific reagents make tree shrews unlikely to become a viable model (**Table 3**).

### MOUSE MODELS

Although mice are naturally resistant to HCV infection, humanized chimeric mice with normal human hepatocytes exhibit prolonged infection following inoculation with HCV (Mercer et al., 2001). This model has been very helpful for preliminary antiviral drug evaluation, but offers little for the study of HCV pathogenesis since the chimeric mice are immunodeficient. Ploss et al. (2009) generated transgenic mice expressing human CD81, scavenger receptor type B class1, claudin 1, and OCLN and infected them with HCV. This humanized model allowed for the investigation of HCV co-receptor biology *in vivo* using antibodies against CD81 and E2 to inhibit viral entry, but was limited by inefficient replication and induction of immune responses against the adenovirus vector, which was used to express the human receptors (**Table 3**).

## CHARACTERISTICS/NATURAL HISTORY OF GBV-B INFECTION

### VIROLOGY

Similar to HCV, GBV-B has a positive-sense single stranded RNA genome and a single ORF encoding the structural (core, E1 and E2 proteins) and the non-structural (p13, NS2, NS3, NS4A, NS4B, NS5A, and NS5B) proteins (Muerhoff et al., 1995). The non-structural proteins are essential for replication, as is the 5′ UTR that directs translation and release of the viral RdRp initiating replication (De Tomassi et al., 2002; Warter et al., 2009). Similar to the p7 protein of HCV, GBV-B also has a 13-KDa protein, p13, located between E2 and NS2 that is essential for viability (Takikawa et al., 2006). Only short regions of nucleotide sequence similarity are shared by GBV-B and HCV including a 130-nucleotide segment of 62% homology in the NS3 region and a 516-nucleotide segment of NS5 of 68% homology (Muerhoff et al., 1995). The polyproteins of HCV and GBV-B have approximately 25–30% homology at the amino acid level, but the 5′ and 3′ UTR are more distinct (Muerhoff et al., 1995; Rijnbrand et al., 2000). The NS2 protein of GBV-B shares membrane topology and has cysteine protease activity similar to HCV (Boukadida et al., 2014). At a functional level, the NS3 protease of GBV-B can correctly process the HCV polyprotein (Scarselli et al., 1997) and HCV/GBVB chimeric NS3 proteins are enzymatically active (Butkiewicz et al., 2000). Interestingly, while both HCV and GBV-B NS5A proteins activate the p13 kinase pathway, HCV NS5A blocks the Ras-Erk pathway, whereas GBV-B NS5A does not (Macdonald et al., 2003, 2004; Mankouri et al., 2008b). Despite an error-prone RdRp, very few amino acid substitutions have been observed, limiting the quasispecies variation of GBV-B in infected animals (McGarvey et al., 2008). Unlike HCV, no hypervariable region was found in the E2 gene of GBV-B in a study by Nam et al. (2004) and McGarvey et al. (2008) calculated the mutation rate of GBV-B to be 0.6 × 10^-3^ nucleotide substitutions/site/year. By comparison, the error rate for HCV RdRp has been estimated at 1.4 to 1.9 × 10^-3^ nucleotide substitutions/genome/site/year (Ogata et al., 1991; Okamoto et al., 1992). These virological differences likely account for the significant differences in quasispecies diversity when comparing GBV-B and HCV.

### PATHOGENESIS

GB virus B is hepatotropic, but has been shown to disseminate to hematolymphoid and genital tissues, including lymph nodes, spleen, PBMC, kidney, testis, and bone marrow (Ishii et al., 2007), much like HCV. The course of experimental GBV-B infection in New World monkeys usually results in acute hepatitis that resolves within 2–3 months post-infection with significant increases in serum enzymes such as ALT and isocitrate dehydrogenase (Lanford et al., 2003; Bright et al., 2004; Weatherford et al., 2009a). GBV-B infection leads to a rapid rise in viremia reaching peak levels of 10^7^–10^10^ genomic equivalents (GEs)/ml by weeks 2–3 and then plateaus before clearance (Jacob et al., 2004; Woollard et al., 2008). Different from HCV, the liver enzyme glutamate dehydrogenase has been shown to be associated with viral clearance and hepatocyte destruction, rather than serum ALT levels (Bright et al., 2004). In liver, degeneration and apoptosis of hepatocytes and disruption and dilation of sinusoids have been observed pathologically in GBV-B infected animals (Martin et al., 2003; Jacob et al., 2004). In a marmoset study by Weatherford et al. (2009a), two disease phenotypes were observed – susceptible and partially resistant. Upon further passaging, the GBV-B virus was able to adapt even in partially resistant animals. GBV-B also infects owl monkeys of the *Cebidae* family but pathogenesis is significantly attenuated as indicated by peak viral titer 2–3 logs lower and milder hepatitis as compared to tamarins and marmosets (Bukh et al., 2001).

### VIRUS PERSISTENCE

A major difference between HCV and GBV-B infections is the chronic nature of HCV (**Table 3**). Generally GBV-B causes an acute infection, although a few cases of chronicity have been reported. Viral persistence for 2 years has been reported in a tamarin infected by intrahepatic inoculation with a synthetic viral RNA before spontaneous resolution (Martin et al., 2003). In another study transfection with a poly (U) tract deletion mutant rendered a tamarin persistently infected until its death at week 90, whereas in other tamarins, acute resolving infection with clearance at week 12 was observed (Nam et al., 2004). In infected marmosets (Jacob et al., 2004), early peak viremia was followed by clearance within 8 weeks in some animals whereas other animals with delayed peak viral load remained viremic up to 6 months. Similarly, Iwasaki et al. (2011) showed that two out of four marmosets infected with a molecular clone, pGBB, developed long-term chronic infection for up to 3 years with recurrent viremia at low levels, similar to chronic infections in chimpanzees. In efforts to increase persistence in the GBV-B model, immunosuppressive drugs have been utilized. Treatment with FK506 in marmosets prior to GBV-B inoculation resulted in higher viral loads and severe liver pathology but did not lead to viral persistence (Jacob et al., 2004). In most infection studies that showed viral persistence, virus from persistently infected animals did not result in chronic infections when transferred to other recipients. This suggests that persistence may be primarily dependent on individual host factors.

### IMMUNOLOGY

One of the first responses to HCV infection in the liver is induction of type I interferons that are thought to aid in viral clearance. However, HCV inactivates early innate antiviral defenses via its NS3/4A protease, which has been postulated to lead to persistence (Foy et al., 2003, 2005; Li et al., 2005; Meylan et al., 2005). Similarly, the GBV-B NS3/4A protease inhibits IFN-inducing pathways, notably RIG-I signaling (Chen et al., 2007). When primary hepatocyte cultures were exposed to IFN-α prior to GBV-B infection, potent antiviral activity blocked virus infection (Chavez et al., 2009). By comparison, in cultures with pre-established GBV-B infection there was reduced antiviral activity, suggesting replicating virus could exert anti-IFN actions.

Much like HCV, T cell immunity has been correlated with viral clearance in marmosets infected with GBV-B (Woollard et al., 2008). Virus-specific T cells were detected in blood at 7 weeks post-infection, coinciding with viral clearance and GBV-B specific IFN-γ responses at week 5 post-infection coincided with a 100-fold reduction in viral load. Virus-specific T cells have also been found to accumulate in the liver of infected marmosets, and the majority of the T cell responses (based on IFN-γ ELIspot) are generated against epitopes in the NS3 and NS4A proteins (Woollard et al., 2008). Upon re-infection of marmosets, the sharp increase in the T cell response suggests the mobilization of memory T cell responses even though no take of infection was observed (Woollard et al., 2008). In marmosets infected with a molecular clone of GBV-B, persistent viremia for 6 months was accompanied by marked influx of MHC class I restricted CD8^+^ T lymphocytes associated with hepatic pathology (Jacob et al., 2004). This is in agreement with studies that have shown predominance of CD8^+^ T cells in the liver during chronic HCV contributing to liver injury (Fiore et al., 1997).

Unlike T cell immunity, the humoral response to GBV-B infection is less well-characterized. Anti-NS3 antibody production peaks toward the end of infection and declines following clearance (Beames et al., 2000). The antibody levels fall rapidly in the absence of viral replication (Beames et al., 2000), which is similar to chimpanzees infected with HCV (Bassett et al., 1998). However, in multiple instances a delayed antibody response has been associated with GBV-B persistence (Martin et al., 2003; Iwasaki et al., 2011).

## GBV-B CHIMERAS

Attempts to improve on the GBV-B model have resulted in construction of chimeric viruses where functional segments of the HCV genome have been incorporated while retaining the ability of GBV-B to replicate and cause liver disease in small primates. These chimeras are extremely valuable for testing candidate therapeutics by targeting specific HCV sequences, but their utility has been marred by difficulties in maintaining efficient take of virus and persistent replication. In HCV infection the IRES directly binds to 40S ribosomal subunit and is a prime candidate target for drug therapeutics. Rijnbrand et al. (2005) constructed a chimeric RNA encoding domain III from the HCV IRES element and while the virus was found to be replication competent, replication levels were generally low and generated adaptive mutations that were necessary for efficient replication. The same chimeric infection in marmosets displayed wide-ranging phenotypes from susceptible to resistant as seen in wild-type GBV-B infection (Weatherford et al., 2009b). Unlike wild-type virus, the chimeric virus failed to adapt in the resistant marmosets on serial passages, indicating loss of fitness (Weatherford et al., 2009b). The first 27 to 31 N-terminal amino acids of the HCV E2 protein hypervariable region 1 (HVR1) have a high degree of sequence variability between different HCV isolates (Weiner et al., 1991; Kurosaki et al., 1994; Alfonso et al., 2004), and have been suggested to contain a dominant neutralizing epitope (Farci et al., 1996). Haqshenas et al. (2007) generated a chimeric GBV-B encoding the HVR1 of HCV and were able to demonstrate successful infection *in vivo*. However, marmosets cleared the virus as early as 3 weeks post-infection and therefore it was not possible to generate anti-HVR1 antibodies in these animals. Recently, Li et al. (2014) generated GBV-B chimeras containing full-length HCV genes of either whole structural core or all envelope proteins. Infected marmosets remained viremic up to 44 weeks and exhibited significant liver pathology and a strong T cell response. Given the restricted tropism of HCV to humans and chimpanzees, the capability of this chimera with HCV envelope proteins to infect marmosets and primary marmoset hepatocyte cultures could suggest conserved viral receptors and virus–host interactions.

## GBV-B DRUG TESTING MODELS

The GBV-B infection model has also been used for the screening of candidate vaccines and other therapeutic agents such as interferon and ribavarin. Ribavarin treatment reduced viral RNA levels by four logs by inducing error prone replication in culture, but *in vivo* treatment showed no obvious reduction in viremia (Lanford et al., 2001). Bright et al. (2004) demonstrated the efficacy of HCV antivirals against GBV-B by showing that HCV NS3 protease inhibitors could block replication both *in vitro* and *in vivo*. Furthermore, a *trans*-lactam, GW0014X was also found capable of preventing GBV-B infection in marmosets. RNA interference, used in gene silencing, has also been applied therapeutically in GBV-B-infected animals. Marmosets administered cationic liposome-encapsulated siRNA (CLsiRNA) directed against GBV-B virus controlled virus replication in manner dependent on CL-siRNA dosing, reaching levels of complete inhibition at 5 mg/kg (Yokota et al., 2007).

## CONCLUDING REMARKS

During the last decade various reports have shown the utility of GBV-B infection as a surrogate model for HCV. Although the GBV-B/marmoset model has a lower chronicity rate, it has a number of advantages over other animal models including lower cost, availability of chimeric viruses, effectiveness of HCV drugs against GBV-B, and a tractable disease course. While the development of new antivirals has changed the landscape of the HCV field, the cost-prohibitive nature of these treatments underscores the ongoing need for a successful vaccine. Thus, we propose that the GBV-B/marmoset model warrants revisiting as a thoughtful research tool for development of immunotherapeutics, new antivirals, and vaccine modalities for eventual translation into human studies.

## Conflict of Interest Statement

The authors declare that the research was conducted in the absence of any commercial or financial relationships that could be construed as a potential conflict of interest.
